# Production of an Antibody Fragment (scFv) Targeting PcrV Protein of *Pseudomonas aeruginosa* in Fed-Batch Cultivation Mode

**DOI:** 10.52547/ibj.25.6.390

**Published:** 2021-10-16

**Authors:** Saba Karam, Mozhgan Raigani, Sahar Hassani Afshar, Yeganeh Talebkhan, Elham Bayat, Samira Komijani, Leila Nematollahi, Farzaneh Barkhordari, Mehdi Shafiee Ardestani, Fatemeh Davami

**Affiliations:** 1 International Campus, School Of Pharmacy, Tehran University of Medical Sciences, Tehran, Iran;; 2 Biotechnology Research Center, Pasteur Institute of Iran, Pasteur Avenue, Tehran, Iran;; 3 Department of Radiopharmacy, Faculty of Pharmacy, Tehran University of Medical Sciences, Tehran, Iran

**Keywords:** Fed Batch, recombinant protein, Pseudomonas aeruginosa, scFv

## Abstract

**Background:**

*Pseudomonas aeruginosa* is one of the opportunistic pathogens causing frequent hospital-acquired life-threatening infections in mechanically ventilated patients. The most significant virulence factor of *P. aeruginosa* is T3SS. PcrV is an important structural protein of the T3SS.

**Methods:**

In the current investigation, a recombinant scFv mAb against the PcrV protein was expressed in EnBase® (fed-batch) cultivation mode. The pETiteTM N-His SUMO Kan vector, including anti-PcrV scFv gene, was transformed into *Escherichia coli* (BL21) cells. The expression and solubility of anti-PcrV scFv protein were investigated at two different temperatures (25 °C and 30 °C) and at different induction times (4, 6, 8, 12, and 24 hours).

**Results:**

Increased efficiency was achieved by EnBase® compared to LB broth; owing to the slow release of glucose, the maximum level of solubility and total protein expression was observed in EnBase® cultivation system at 30 °C and 24 h post induction. Furthermore, IC_50_ for anti-PcrV scFv protein was determined to be approximately 7 μg/mL.

**Conclusion:**

Anti-PcrV scFv produced in this study showed promising *in vitro* results, protecting RBC from lysis by* P. aeruginosa* (*exoU+*).

## INTRODUCTION

*Pseudomonas aeruginosa*, a human Gram-negative bacterium is found in healthy individuals^[^^1^^]^. Instead, it often colonizes immunocompromised patients, including those with cystic fibrosis, cancer, or acquired immunodeficiency syndrome^[^^2^^]^. This bacterium is the main cause of nosocomial infections, particularly in mechanically ventilated patients^[^^3^^]^. Also, it complicates 90% of cystic fibrosis deaths because of its resistance to many effective antibiotics^[^^4^^]^. Over the recent years, antibiotic-resistant infections have been recognized as a major issue in hospitalized patients; therefore, exploring recombinant antibody against *P. aeruginosa* may contribute to the prevention or treatment of its serious infections^[^^5^^]^.

In *P. aeruginosa*, the same as most other Gram-negative pathogens, T3SS is utilized to inject bacterial toxins directly into the cytoplasm of target cells^[^^6^^,^^7^^]^. This system involves various proteins, some of which are assembled into a "secretory" structure including a "needle" through which effector molecules travel target cell^[^^8^^,^^9^^]^. A complex regulatory mechanism is involved in controlling effector molecule injection, an essential component of which is PcrV, a 32-kDa protein^[^^10^^]^. Due to the appropriate immune response against PcrV, it is a proper target for the production of mAbs^[^^11^^]^. The desired whole and fragment anti-PcrV mAb has been engineered to inhibit the PcrV protein of T3SS, suggesting them as prophylactic mAbs.

To surmount some fundamental limitations of full-size mAbs such as low tissue penetration, limited range of possible targets, and, importantly, high production costs, attempts have been made to produce antibody fragments with smaller size including scFvs. The scFv has several benefits, such as greater tumor penetration, more rapid blood clearance, lower retention times in non-target tissue, and reduced immunogenicity^[^^12^^]^. The Fab of anti-PcrV have previously been investigated^[^^13^^]^, but no report on anti-PcrV scFv is available. In this study, a recombinant scFv of anti-PcrV was analyzed for the first time.

In accordance with the Southeast Collaboratory for Structural Genomics reports, soluble form of protein accounts for 22.9% of *E. coli *expressed proteins*. *Different strategies have been applied to enhance both expression levels and solubility of desired recombinant proteins. The use of weaker promoters^[^^14^^]^, co-expression with foldases and chaperones^[^^15^^]^, using lower temperatures^[^^16^^]^ and protein fusion tags such as SUMO^[^^17^^]^, as well as optimizing the cultivation conditions^[^^18^^]^ are a number of these methods. 

SUMO is a small protein of ~11kDa derived from the yeast. It is a kind of as a post-translational modification fused to the N-terminus of the recombinant protein to improve the expression as well as solubility of the produced protein in prokaryotic and eukaryotic cells^[^^19^^]^. SUMO tag can further improve protein folding and facilitate the purification steps by 6-His motif at its amino terminus. Following the expression and purification of the fusion protein, the SUMO-tag is cleavable by specific (SUMO) proteases to promote the native protein structure^[^^20^^]^. 

Media optimization techniques such as EnBase®, a substrate auto-release system, are based on the controlled glucose release into the cultivation medium using the enzymatic degradation of carbon source^[^^21^^]^. In the EnBase® cultivation system, the enzymatic glucose release system provides high cell densities, high protein yields, and a noticeably enhanced proportion of soluble proteins in the harvested cells^[^^22^^]^.

In this study, the soluble scFv of anti-PcrV was expressed in *E. coli* for the first time. We further investigated the effect of EnBase® fed-batch cultivation mode to enhance the expression of anti-PcrV scFv protein. To evaluate the influence of temperature reduction and induction time on the solubility and productivity of the recombinant antibody, two different temperatures (25 °C and 30 °C) and various induction times (4, 6, 8, 12, and 24 hours) were analyzed. Finally, the protective effects of anti-PcrV scFv against *in vitro* infection of *P. aeruginosa* were evaluated.

## MATERIALS AND METHODS

**Anti-PcrV scFv gene cloning**

The gene sequences encoding scFv against *P. aeruginosa* PcrV were designed. The sequence for the anti-PcrV scFv was designed based on a literature review in search for the best active anti-PcrV antibody in the time of performing the project design. We used the VH and VL sequences based on V2L2 mAb published by Warrener *et al.* 2014^[^^25^^] ^and patent number US 2015 0023966 both from MedImmune, USA. The final format of scFv was N-VH-(G4S)4 linker-VL-C terminal with the following sequence:

EMQLLESGGGLVQPGGSLRLSCAASGFTFSSYAMNWVRQAPGEGLEWVSAITISGITAYYTDSVKGRFTISRDNSKNTLYLQMNSLRAGDTAVYYCAKEEFLPGTHYYYGMDVWGQGTTVTVSSGGGGSGGGGSGGGGSGGGGSAIQMTQSPSSLSASVGDRVTITCRASQGIRNDLGWYQQKPGKAPKLLIYSASTLQSGVPSRFSGSGSGTDFTLTISSLQPEDFATYYCLQDYNYPWTFGQGTKVEIK. This sequence was back-translated to DNA and codon optimized based on E. coli codon bias and synthesized commercially (Generay Biotech [Shanghai] Co., Ltd). The codon optimized sequence of Anti-PcrV scFv was then PCR amplified using forward and reverse primers designed based on protocol described in Lucigen kit (Lucigen Corporation, USA). The PCR product, a bond of 1226 bp, was directly transformed into HI-Control 10G competent cells along with the pETite N-His SUMO Kan vector by the heat shock method. 

**Screening for recombinant bacteria**

The plasmid extraction was accomplished according to the instruction manual provided by Yekta Tajhiz Plasmid DNA extraction mini Kit (Tehran, Iran). Colony PCR were performed on extracted plasmids by the SUMO forward and pETite reverse primers and also by specific primers included in the kit. The amplified DNA was analyzed by agarose gel electrophoresis.

**Protein expression**

The transformed* E. coli* HI-control BL21 (DE3 Strain) cells were cultured in the EnBase® medium (BioSilta Oy, Oulu, Finland) as mentioned below. For batch mode cultivation experiment, the single recombinant *E. coli* (BL21 strain) colonies were used to inoculate LB-kanamycin (30 μg/mL) broth and were shaken with 180 rpm at 37 °C for 3–4 h. After the culture optical density reached 0.5-0.6 at 600 nm, the expression was induced by the addition of 1 mmol/L IPTG. Bacterial pellet collection was carried out at 4, 6, 8, and 24 h following incubation.

**SDS-PAGE and Western blot analysis**

SDS-PAGE and Western blot analysis were used to confirm the expression of antibody against PcrV. Induced recombinant cells were harvested and analyzed by electrophoresis on a 12% SDS-PAGE with Coomassie Brilliant Blue staining method according to the Laemmli^[^^23^^]^. Western blotting was carried out based on Sambrook* et al.*^[^^24^^]^. The anti-His antibody at a dilution of 1/500 in PBS (Abcam, USA) and horseradish peroxidaseconjugated goat anti-rabbit antibody (1/2500 dilution, Santa Cruz. USA) were applied as primary and secondary antibodies, respectively. Protein band visualization was performed by adding the 3,3′-diaminobenzidine solution (Sigma-Aldrich, Germany). A his-tag fused, 40 kDa protein, developed in our lab was used as the positive control. 

**Effects of different cultivation mode and temperatures on anti-PcrV antibody expression/ solubility**

Based on Biosilta kit protocol for fed-batch EnBase® cultivation, white tablets, including culture media components, were solved in sterile water for preparation of starter medium, the recombinant single colonies were inoculated in LB (5 mL), containing kanamycin (30 μg/mL), and incubated at 37 °C for 6–8 h, until OD_600_ of 0.6 was achieved. Subsequently, 2 mL of the starter was added to the 50 mL of prepared EnBase® culture medium, comprising kanamycin (30 μg/mL) and reagent A (25 μL). The growth media was incubated in a shaker incubator (180 rpm) overnight. After15–18 h, the grown culture (1 mL) was gathered prior to sample induction. Based on manufacturer’s protocol, a booster tablet, containing extra nutrients and polysaccharide substrate, was added in this step. Moreover, 50 μL of 600 IU Reagent A and IPTG (at final concentration of 1 mmol/L) were further added to the cultivation media. Sample collection was performed at 4, 6, 8, 12, and 24 h post induction. Finally, the bacterial pellets were recovered by centrifugation and kept at -20 °C until later analysis. The expression of anti-PcrV scFv was investigated at two different temperatures (25 and 30 ºC) in order to evaluate the effect of reduced temperature on the solubility of recombinant antibody. Actually, as there was no temperature shift in EnBase protocol, the whole process was performed in different temperatures.

**Analysis of soluble expression**

To evaluate the soluble fraction of recombinant anti-PcrV scFv, bacterial pellets were re-suspended in Tris-EDTA buffer and sonicated for eight cycles, with pulses of 30 s and intervals of 20 s. The sonicated samples were centrifuged at 4 °C at 15,000 ×g for 20 min. The clear supernatant was retained as a soluble fraction. Protein concentrations were determined by a NanoDrop spectrophotometer using absorbance at 280 nm. SDS-PAGE was performed for total, pellet, and supernatant fractions of different experiments. 

**Recombinant protein purification**

The puriﬁcation procedure was performed in conformity with Agarose Bead Technologies based on Ni-NTA affinity chromatography protocol. In order to solubilize the inclusion bodies, the cell pellets from an *E. coli* expression culture were thawed on ice. Thereafter, 1 g of pelleted wet cells was resuspended in 5 mL of binding buffer (50 mM of NaH_2_PO_4_, 300 mM of NaCl, and 10 mM of imidazole; pH 8.0). The suspension was then sonicated on ice. Following centrifugation, the pellet was kept on ice to collect the inclusion bodies. The pellet was resuspended in the aforementioned binding buffer. After centrifuging, 2.0 mL (per g wet cells) of the same buffer containing 8 M of urea, was added. The inclusion bodies were dissolved by stirring on ice for 60 min. In order to eliminate the insoluble material, the solution was centrifuged, and the supernatant was collected. The recombinant protein was purified using a Ni-NTA column. The content of wash and elution buffers were the same, including 50 mM of NaH_2_PO_4_, 300 mM of NaCl, and 8 M urea (pH 8.0), except for imidazole, which was 20 mM for elution buffer and 250 nM for wash. 

**RBC lysis inhibition test**

RBCs were prepared from fresh whole blood obtained from healthy volunteers by centrifugation and multiple PBS washes. RBCs (2% [v/v] final) washed in normal saline (0.9), and anti-PcrV scFv supernatant in PBS was applied into a 96-well plate. *P. aeruginosa *was grown in 5 mL of yeast extract-tryptone medium (Difco, USA) to mid log phase, harvested and resuspended in PBS at an OD_600_ of 0.15^[^^25^^]^. Equal amounts of bacterial suspension (10 µL) was added to the RBC-antibody mixture (the concentration gradient of antibody was considered), mixed and incubated at 37 °C for 2 h. Brief centrifuge was applied to the plates in order to pellet the intact RBCs. The supernatants were then transferred to a 96-well plate for the measurement of OD 405 nm wavelength.

## RESULTS

**Validation of expression cassette**

**Fig.1:**
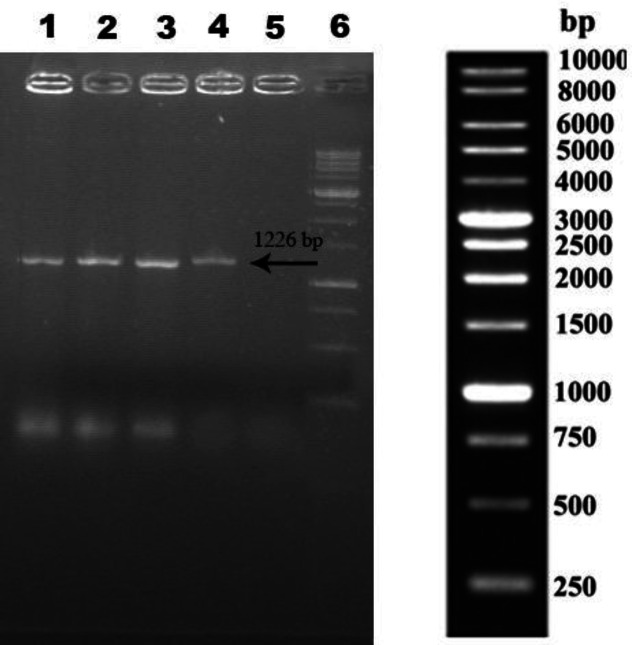
PCR analysis of extracted recombinant plasmids from kanamycin-resistant *E. coli *cells. PCR products were electrophoresed on a 1% agarose gel. Lanes 1–4, amplification of anti-PcrV scFv gene; lane 5, negative control; lane 6, 1-kb DNA markers

The cloning of anti-PcrV scFv gene fragment in pETite N-His SUMO Kan vector was confirmed by colony PCR. Figure 1 illustrates the results of gel electrophoresis; The PCR with expected size of 1226 bp was observed.

**Analysis of expression via** **SDS-PAGE and Western blotting procedure**

**Fig. 2:**
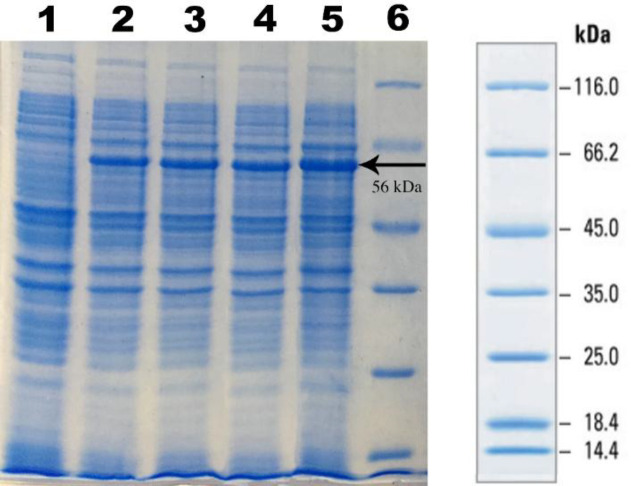
SDS-PAGE analysis of anti-PcrV scFv expression in LB medium. Lane 1, cell lysates before IPTG induction; cell lysates 4 h (lane 2), 6 h (lane 3), 8 h (lane 4), and 24 h (lane 5) after induction; lane 6, Fermentas protein marker SM0671

**Fig. 3:**
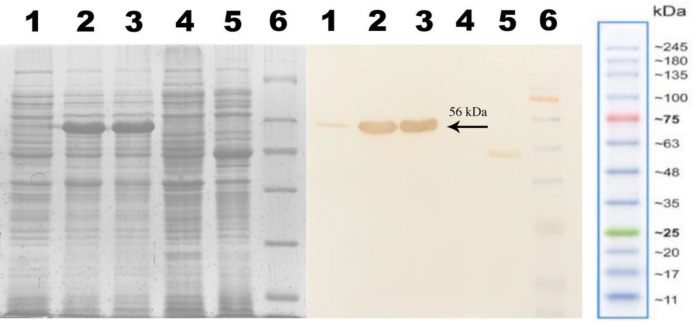
Western blotting analysis of the recombinant 6-His SUMO tag anti-PcrV scFv protein. Lane 1, cell lysates before IPTG induction; lanes 2 and 3, the 56 kDa 6-His SUMO tagged anti-PcrV scFv protein; lane 4, negative control; lane 5, a 40-kDa 6-His tagged protein as the control; lane 6, protein marker

N-His SUMO-anti-PcrV protein with a predicted molecular weight of 58 kDa was analyzed via gel electrophoresis on 12% agarose gel (Fig. 2). According to Western blotting, as shown in Figure 3, the anti-His IgG was able to detected the target band, confirming the expression of anti-PcrV scFv protein in *E. coli *cells. No reacting band was found in the protein analysis of the negative control.

**Fed-batch cultivation mode effects on the expression and solubility of anti-PcrV scFv**

**Fig. 4:**
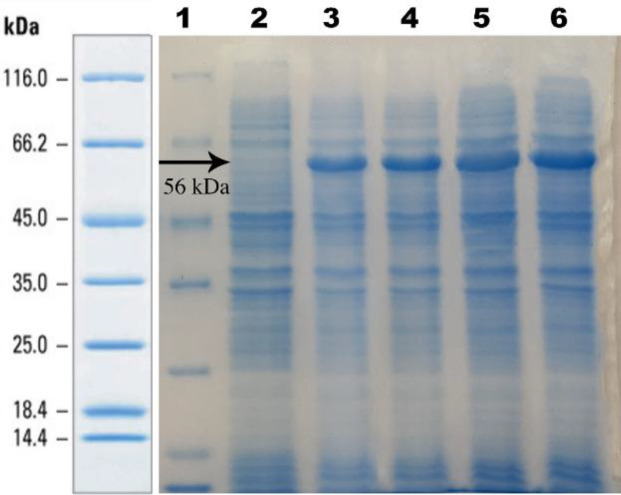
SDS-PAGE analysis of expressed recombinant SUMO anti-PcrV scFv in EnBase® medium at different times. Lane 1, fermentas protein marker SM0671; lane 2, cell lysates before IPTG induction; cell lysates 4 h (lane 3), 6 h (lane 4), 8 h (lane 5), and 24 h (lane 6) after induction

In order to investigate the effect of cultivation mode on expression yield at fed-batch process and conventional LB medium, the recombinant *E. coli *HI-control 10G cells were induced by IPTG at 25 °C in both growth media, and the pellets were collected at 0–24 h following induction. The total anti-PcrV scFv expression was inspected on a 12% resolving gel. Our results showed that the anti-PcrV scFv protein was successfully expressed in EnBase® fed-batch system and LB medium, which did not significantly differ (Fig. 4).

**Effect of temperature reduction on the expression and solubility of anti-PcrV scFv**

**Fig. 5:**
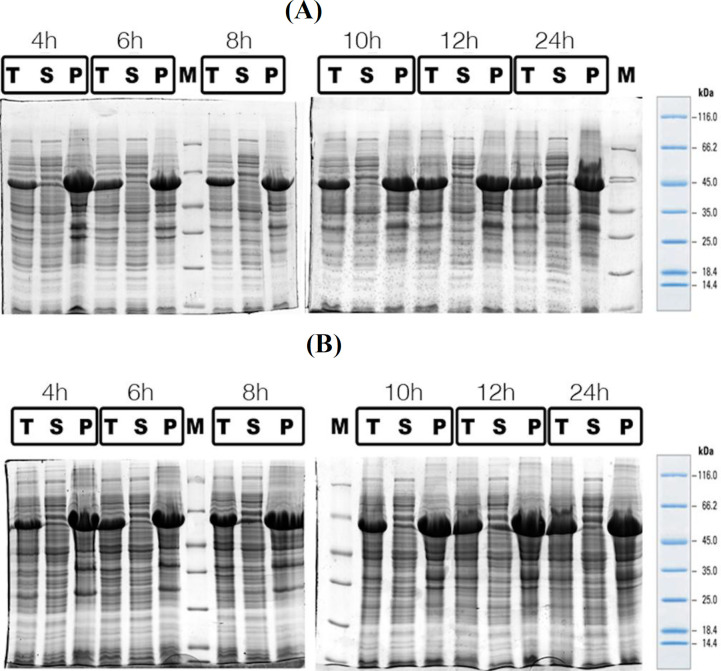
SDS-PAGE analysis of soluble and insoluble expression of recombinant SUMO anti-PcrV scFv expression in EnBase® medium at different temperatures and times. The distribution of total cell lysates, pellet, and soluble fractions during different time intervals post IPTG induction. Coomassie stained gel under reduced condition. The distribution of total cell lysates, pellet, and soluble fractions in EnBase® medium during different time intervals post IPTG induction at (A) 25 °C and (B) at 30 °C. T, total lysate; S, supernatant or soluble fraction; P, pellet; M, Fermentas protein marker

The total and soluble expression of anti-PcrV scFv was assessed in two temperatures (30 and 25 °C). In order to find the optimum time length for induction, the recombinant bacteria were induced for different hours (4, 6, 8, 12, and 24) at any of the selected temperatures. Using SDS-PAGE technique, the amount of recombinant SUMO anti-PcrV scFv protein was analyzed in both the total pellet and supernatant. According to the Figure 5, the culture temperature (30 °C) resulted in the optimum protein expression using EnBase® cultivation system. Based on the results, most of the anti-PcrV scFv proteins expressed at both temperatures were formed as inclusion bodies. The maximum solubility and total protein expression obtained for anti-PcrV scFv protein was observed at 24 h post IPTG induction time at 30 ºC.

**Purification of anti-PcrV scFv**

**Fig. 6:**
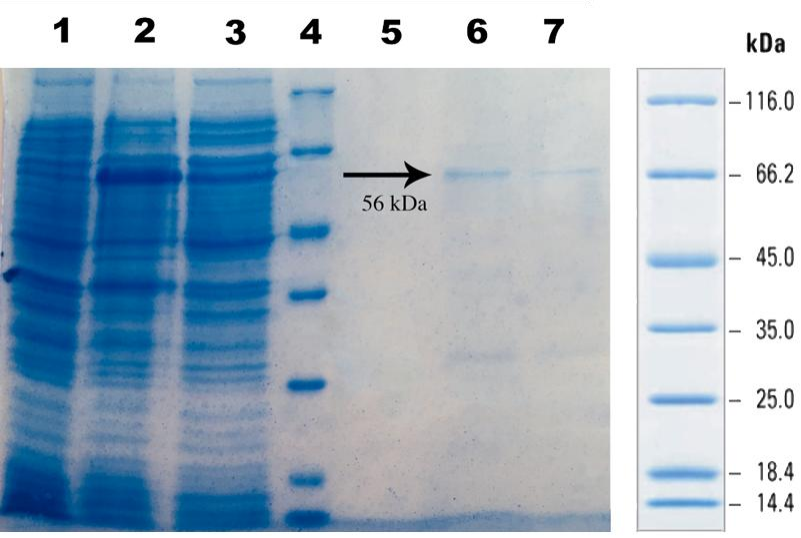
The SDS-PAGE analysis of the Ni-NTA affinity chromatography of the recombinant SUMO anti-PcrV scFv protein. Cell lysates before (lane 1) and after (lane 2) IPTG induction; lane 3, flow-through; lane 4, Fermentas protein marker; lane 5, wash sample; lanes 6 and 7, the fractions of elution buffer

As shown in Figure 6, the anti-PcrV scFv protein was purified by the Ni-NTA column in order to analyze the biological activity (RBC lysis inhibition). The purified protein demonstrated as a 56-kDa band in elution fractions post affinity purification.

**Analysis of RBC lysis inhibition test (activity)**

RBCs were incubated with log-phase *P. aeruginosa *and 0, 0.1, 0.2, 2, 4, 10, 20, 21.2, 26.5, 31.8, 37.1, and 42.4 μg/mL of purified anti-PcrV scFv (as the inhibitor of RBC lysis). Following lysis, the hemoglobin was released, and supernatants were measured by a spectrophotometer at 405 nm. The percentage of lysis inhibition was measured by comparing the OD values of the test wells with those of the control wells, which received no anti-PcrV scFv. The results showed that in the absence of recombinant protein, RBC was completely lysed by *P. aeruginosa* (*exoU+*). Triton X-100 (1) was used as a positive control. By increasing the antibody concentration in each well, the percentage of RBC lysis declined. Therefore, IC_50_ for anti-PcrV scFv protein was determined to be at around 7 μg/mL of scFv. Further increase in the concentration of antibody resulted in no significant increment in the cellular protection. The results also indicated that the expressed recombinant proteins were able to protect RBC from lysis by* P. aeruginosa* (*exoU+*).

## DISCUSSION

The high specificity of mAbs and their functional derivatives have led to the selective treatment of diseases with less side-effects and no harm to the beneficial host bacteria^[^^26^^]^. Antibody fragments (scFv herein), which have myriad advantages compared to whole mAbs, could be made by reducing the size of whole mAb to enhance tissue penetration. However, the half-lives of antibody fragments are lower than the whole antibody, and to enhance their half-life, soluble monomeric IgG1 CH3 has been suggested as a new scaffold^[^^27^^]^. Monomeric IgG1 CH3 exhibiting pH-dependent binding to neonatal Fc receptor (FcRn) is a promising fusion partner for therapeutic proteins with increased therapeutic efficacy. 

By development the recombinant antibody technology, the expression techniques are expanded to ameliorate the quality and performance of the desired products^[^^28^^]^. Among all the expressive systems, *E. coli *is a prokaryotic host for producing high amounts of recombinant proteins and antibody fragments such as Fabs^[^^29^^]^. Noteworthy, this host still has certain weaknesses in expressing complex molecules due to the lack of post-translational modification and soluble expression^[^^30^^]^.

Based on Figure 5, the expression of anti-PcrV scFv was evaluated at 25 °C and 30 °C. Sample collection was performed 0–24 h after induction by IPTG. The optimum yield of total protein was achieved 24 h post induction at 30 °C. Additionally, the maximum level of soluble anti-PcrV scFv protein, was also observed at 24 h post induction time in EnBase® cultivation medium at 30 °C. This incident may be explained by the fact that enzymatic glucose release system was enhanced by higher temperature^[^^22^^]^. Therefore, at high temperatures, the amount of proteins can be increased compared to low temperatures. Furthermore, with EnBase® cultivation, promising conditions for long-term induction and controlled growth were found at 30 °C, owing to higher oxygen solubility and less evaporation^[^^22^^]^. On the other hand, at low temperatures, the high levels of recombinant proteins were expressed by inappropriate folding, possibly ensuring the solubilization of recombinant proteins^[^^22^^]^. A similar outcome was reported by Namvar and colleagues^[^^18^^]^ who expressed α-Luffin protein in a fed-batch-based cultivation system and a SUMO fusion tag. They demonstrated that the highest level of total expression and soluble form in EnBase® cultivation system was achieved at 12 and 24 h post induction time at 25 °C and 30 °C, respectively. As illustrated above, the best titer of soluble protein fraction was obtained at 24 h post induction time at 30 °C in EnBase® cultivation medium, which is in line with Namvar *et al.*’s^[^^18^^]^ study, possibly because of the chaperone resembling feature of SUMO. In contrast to the survey conducted by Namvar *et al.*^[^^18^^]^, Yun and co-workers^[^^31^^]^ demonstrated that in terms of anti-PcrV scFv, the best protein expression was observed at 30 °C.

The EnBase® fed-batch-like medium has been effectively used for high-yield cytoplasmic expression of several biologically active recombinant proteins^[^^30^^]^. According to Figure 4, the anti-PcrV scFv protein was successfully expressed in the EnBase® fed-batch system and LB medium, which did not significantly differ. The EnBase® Flo cultivation system in shaken cultures was also exerted by Krause *et al.*^[^^22^^]^ who provided elevated cell densities without distinguishing reduction in the productivity per cell and improved yield of soluble recombinant proteins compared to commonly used media. In another study by Secher *et al.*^[^^32^^]^ the effectiveness of an anti-P*. aeruginosa* serotype O11 lipopolysaccharide IgM/κ mAb (panobacumab) was investigated in a neutropenia murine model. They showed that panobacumab enhanced the removal of *P. aeruginosa* in neutropenic hosts, similar to when used in combination with antibiotics in immunocompetent hosts. Panobacumab is directed against the Lipo Poly Saccharide, O-polysaccharide moiety of *P. aeruginosa* serotype IATS O11. They also generated neutropenic mice and investigated the *in vivo* effects of panobacumab on pneumonia caused by *P. aeruginosa*. 

**Fig. 7:**
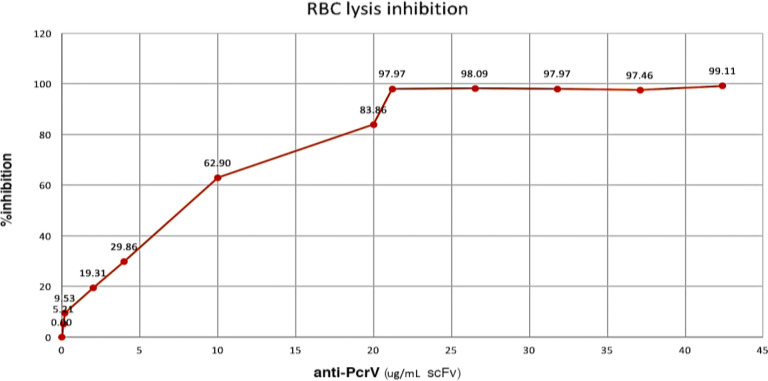
The percentage of *in vitro* inhibition of T3SS by anti-PcrV scFv. RBCs were incubated with log-phase *P. aeruginosa *in the presence of a certain amount of purified anti-PcrV scFv. Spectrophotometry was performed to understand the hemoglobin release profile in the supernatants

Herein, the *in vitro* effects of anti-PcrV scFv were investigated. A similar study was conducted by Ali *et al.*^[^^33^^]^, investigating the efficacy of the anti-PcrV and anti-Psl bispecific human IgG1κ mAb, MEDI3902, in the prevention of nosocomial *P. aeruginosa* pneumonia in high-risk patients. MEDI3902 was found to have appropriate anti-*P. aeruginosa* activity, and the safety and the tolerability profile of this antibody was suitable for use in ventilated ICU subjects. MEDI3902 binds to both the *P. aeruginosa* PcrV protein involved in host cell cytotoxicity and also to the Psl involved in *P. aeruginosa *colonization and tissue adherence. According to Figure 7, the produced anti-PcrV scFv was able to inhibit red cell lysis in laboratory conditions at 21 μg/mL. By increasing the antibody concentration in each well, the percentage of RBC lysis showed a stationary profile. Therefore, IC_50_ was determined to be approximately 7 μg/mL for anti-PcrV scFv protein. The results showed that the expressed recombinant proteins were able to protect RBCs from lysis by* P. aeruginosa* (*exoU+*). In a previous study reported by Warrner *et al.*^[^^25^^]^, the relative T3SS inhibitory activities of V2L2MD and MAb166.2a mAbs were displayed by RBC lysis inhibition assay. They showed that the 50% repressive concentrations were 0.37 μg/mL and 3.7 μg/mL, for V2L2MD and MAb166.2a respectively. In the present study, the IC_50_ dose for our protein was around 7 μg/mL. In another study by Goure and colleagues^[^^34^^]^, the antibody concentrations at 0.25 ng/mL were able to reduce *P. aeruginosa*-induced lysis by 50%. They found that an increase in the concentration of antibodies up to 10 ng/mL in the infection assay completely protected RBCs from lysis. Similarly, Frank *et al.*^[^^35^^]^ explored that the addition of murine MAb 166 IgG to the bacterial inoculums or intraperitoneal transfer to mice and investigated the rate of the survival of infected mice. They reported that higher doses (10 and 50 µg) of purified MAb, caused 80% and 90% survival, respectively. Different IC_50_ concentrations achieved in our study can be explained by the fact that the protein analyzed in the present study was the scFv of mAb with a lower binding activity in comparison to a Fab.^[^^36^^]^. Singh^[^^37^^]^ showed that Fab with lower K_D_ value evinced better binding (1.5 fold higher) with the studied protein as compared to scFv. In addition, the results reported by Maruta *et al.*^[^^38^^]^ suggested that the binding activity of antibody fragments, especially scFv (higher K_D_), might be slightly reduced in comparison to that of whole antibodies. Therefore, reducing the binding activity of anti-PcrV scFv might decrease its efficacy, as seen in Qi *et al.*'s^[^^39^^]^ study in which anti-IL-1-scFv treatments resulted in significantly lower efficacy compared to anti-IL-1-Fab and anti-IL-1-full-length antibody therapies. Further investigation is required in mouse models.

In conclusion, owing to the slow release of glucose, a higher anti-PcrV scFv expression and solubility was achieved in EnBase® cultivation mode, compared with common methods of cultivation. Besides, our results revealed that for this specific protein, increasing the induction temperature to 30 °C at 24 h following induction time, enhanced the expression and solubility of recombinant anti-PcrV scFv protein in the fed-batch cultivation system. We also demonstrated that the antibody against PcrV of *P. aeruginosa* can prevent RBC lysis, thereby likely protecting against the infections of *P. aeruginosa*. 

## CONFLICT OF INTEREST.

None declared.
